# Anti-modified citrullinated vimentin antibody: a novel biomarker associated with cardiac systolic dysfunction in patients with rheumatoid arthritis

**DOI:** 10.1186/s12872-020-01676-x

**Published:** 2020-08-26

**Authors:** Somayye Norouzi, Ali Javinani, Arya Aminorroaya, Maryam Masoumi

**Affiliations:** 1 Department of Internal Medicine, Qom University of Medical Sciences, Qom, Iran; 2 Rheumatology Research Center, Tehran University of Medical Sciences, Tehran, Iran; 3 Tehran Heart Center, Tehran University of Medical Sciences, Tehran, Iran; 4 Clinical Research Development Center, Shahid Beheshti Hospital, Qom University of Medical Sciences, Azadegan Sq., Shahid Beheshti Blvd, Qom, Iran

**Keywords:** Autoantibodies, Anti-citrullinated protein antibodies, Anti-modified citrullinated vimentin, Anti-MCV, Arthritis, rheumatoid, Rheumatoid factor, Ejection fraction, Echocardiography

## Abstract

**Background:**

Studies have demonstrated that seropositive patients with rheumatoid arthritis (RA) are susceptible to cardiovascular diseases (CVDs). In this study, we aimed to determine the association of autoantibodies with the echocardiographic parameters of systolic and diastolic dysfunction in such patients.

**Methods:**

In this cross-sectional study, we evaluated patients with RA who were referred to our clinic from October 2017 to August 2018. After the exclusion of patients with concomitant CVD, all patients underwent transthoracic echocardiography and measurement of plasma autoantibodies. Moreover, possible confounders—including medications, CVD risk factors, Framingham risk score, disease activity score-28, duration of disease, simple disease activity index, and functional status—were assessed.

**Results:**

We studied 135 patients with RA (mean age = 52.3 years; 111 (82.2%) females). We had missing data rates of up to 8.9% for some characteristics. E velocity was inversely correlated with rheumatoid factor (*P* = 0.009). Furthermore, the plasma levels of anti-citrullinated protein and anti-modified citrullinated vimentin (anti-MCV) antibodies were negatively correlated with left ventricular ejection fraction (LVEF) (*P* = 0.019 and *P*<0.001, respectively). After an adjustment for possible confounders, the linear regression model demonstrated that the anti-MCV level and the patient’s age are significant predictors of LVEF. The receiver operating characteristic curve showed that anti-MCV antibody titer≥547.5 (IU/mL) signifies reduced LVEF (<50%) with a sensitivity of 85.7% and specificity of 93% (C-statistic = 0.843).

**Conclusions:**

Our findings showed a significant inverse correlation between anti-MCV antibody titer and LVEF. These results indicate that the application of anti-MCV is promising for the screening and early detection of cardiac systolic dysfunction. Future prospective studies will determine its role.

## Background

Rheumatoid arthritis (RA) is an auto-immune disorder that is mainly characterized by chronic synovitis and the progressive destruction of the joints. RA can involve nearly every organ in the body at a low prevalence when compared to its articular manifestations. According to the high prevalence and chronicity of RA, it has a significant socioeconomic burden [1]. The global disability-adjusted life year of RA has increased over the past decades, with approximately 3.5 million years in 2017, 0.14 of the global share [2]. Introducing novel diagnostic biomarkers, imaging studies, and effective medications have diminished the debilities of the disorder in the face of its increased prevalence [1].

The all-cause mortality rate of RA patients is significantly higher than that of the healthy population [1]. Most recent population-based studies have demonstrated that cardiovascular diseases (CVDs) are the leading cause of death among patients with RA [3, 4]. This can be attributed to the higher prevalence of conventional CVD risk factors among this population. In addition, certain RA-specific risk factors, including systemic inflammatory responses and cytokines, can worsen endothelial cell dysfunction and favor the formation of atherosclerotic plaques [5]. Moreover, the post-transcriptional modification of proteins, including citrullination, exposes some neo-antigens to the immune system. Anti-citrullinated protein antibodies (ACPAs) can react with these neo-antigens and activate an inflammatory cascade, thereby causing tissue damage [6].

According to the high morbidity and mortality of CVD among patients with RA, the European League Against Rheumatism (EULAR) recommended regular assessments of patients in this regard [7]. According to this guideline, symptoms such as prolonged disease duration, extra-articular manifestations, and positive ACPA and rheumatoid factor (RF) were considered as risk factors of CVD. Risk assessment for CVD can be done by certain clinical, laboratory, and imaging studies. Transthoracic echocardiography (TTE) is a reliable, non-invasive, and inexpensive method that can present signs of previous ischemia, myocardial systolic dysfunction, myocardial diastolic dysfunction, and valvular and pericardial abnormalities [8]. Owing to the high prevalence of echocardiographic markers of diastolic dysfunction among patients with RA, TTE seems to be valuable for the early detection of occult CVD [9].

As mentioned previously, seropositive RA patients are susceptible to CVDs. Moreover, RA-specific autoantibodies, especially ACPA, can trigger an inflammatory cascade against the citrullinated neo-antigens. Because patients with RA interstitium of the myocardium have high levels of citrullinated proteins compared to healthy individuals [10], these targeted inflammatory cascades could lead to ventricular systolic and diastolic dysfunction. In the present study, we have aimed to shed light on the correlation between RA-specific autoantibodies and TTE parameters. Moreover, we have investigated the correlation between anti-modified citrullinated vimentin (anti-MCV) antibody titer with ventricular dysfunction parameters, which has not been reported previously.

## Methods

### Study population

We conducted this cross-sectional study on patients with RA from our outpatient rheumatology clinic of Shahid Beheshti hospital, Qom University of Medical Sciences. Patients were selected by a simple random sampling approach, and all participants fulfilled the American college of rheumatology (ACR)/EULAR 2010 RA classification criteria [11]. Patients with a known history of overt CVDs, including coronary artery disease, peripheral arterial disease, cerebrovascular disease, dysrhythmia, and heart failure, were excluded from the study. Moreover, patients receiving biological treatment and cardiotoxic agents (cyclophosphamide, anthracyclines, and illicit drugs) were excluded. In cases of an indication of the primary prevention of CVDs with statins, patients were prescribed these medications. Furthermore, patients with hypertension were treated with anti-hypertensive medications. It should be noted that the participants did not receive any other cardiovascular medications like aspirin, nitrates, or beta-blockers. The study was performed from October 2017 to August 2018.

### Study design

This paper investigated the connection between RA-specific autoantibodies and TTE variables regarding systolic and diastolic dysfunction. In order for the scientific results to be more accurate, we have considered certain confounding factors in our analyses that could independently influence the ventricular function and subsequently alter the TTE findings. The Framingham risk score was used to represent the traditional risk factors for CVD, including sex, age, smoking status, diabetes mellitus history, hypertension, and dyslipidemia [12]. The Framingham risk score was calculated using an online calculator (https://reference.medscape.com/calculator/framingham-cardiovascular-disease-risk). Patients were considered as having dyslipidemia if they met one of the following criteria: 1) use of lipid-lowering agents; 2) total cholesterol≥200 mg/dL; 3) low-density lipoprotein cholesterol≥100 mg/dL; 4) high-density lipoprotein≤40 mg/dL for men or 50 mg/dL for women; 5) triglyceride≥150 mg/dL. Hypertension was defined as systolic blood pressure ≥ 140 mmHg or diastolic blood pressure ≥ 90 mmHg. Moreover, the chronic inflammatory state of RA has a significant effect on a patient’s cardiovascular risk profile. Not only the disease activity at the time of the study is a major contributor, but also the previous sequels of the disorder have noticeable effects on the cardiac system. Therefore, we have calculated the disease activity at the time of the study by considering the disease activity score-28/erythrocyte sedimentation rate (DAS-28/ESR) and a simple disease activity index (SDAI) for RA. According to these indices, all patients were in the remission phase during the study period. The ACR functional class of RA was calculated for considering sequels of the disease. The ACR functional classes demonstrate that the disability of patients occurred over years of suffering from RA. Among our patients with prolonged RA (disease duration≥10 years), 38.5% had an ACR functional class of I, which means that they were able to perform everyday activities without any limitations.

### Laboratory data

Blood sampling was done at the time of the study. Similar laboratory kits were used to accurately assess the association of autoantibodies’ quantitative titer with TTE variables. RF was measured by the agglutination method with a ENISON RF kit. ACPA and anti-MCV antibodies were measured with a CHORUS instrument via an enzyme-linked immunoabsorbent assay and using CHORUS ACPA kit and ORGENTEC anti-MCV kit, respectively. The upper limits of ACPA and anti-MCV antibody measurements were 300 IU/mL and 1000 IU/mL, respectively. ACPA titer up to 5 IU/mL was considered normal, and anti-MCV antibody titer≥20 IU/mL was regarded as positive. In our study population, the RF, ACPA, and anti-MCV were positive among 40.0%, 57.8%, and 46.7% of the patients, respectively. ESR was also measured using the Westergren method and an IMPROVE autoanalyzer. Lipid profiles and fasting blood glucose levels were obtained from the patients’ documents. These had been measured maximally 1 year prior to the study period. All the investigations were conducted in the hospital laboratory without any missing data.

### TTE

All participants underwent TTE and tissue Doppler imaging, which was performed by an expert cardiologist (SN). The TTE was done by a commercially available instrument and a 2.5 MHz probe. The study was performed on the left lateral decubitus position. We evaluated parameters related to cardiac systolic and diastolic dysfunction, including the left ventricular ejection fraction (LVEF), pulmonary arterial pressure (PAP), E velocity, E/e’ ratio, tricuspid annular plane systolic excursion (TAPSE), isovolumic relaxation time (IVRT), and right ventricular systolic motion (RVSm). The LVEF was primarily determined visually, and abnormal values were rechecked via the Simpson method. Among our RA patients, 7 cases (5.2%) had systolic dysfunction with an LVEF of less than 50%.

### Statistical analysis

Data analysis was conducted using SPSS software (IBM Corp. Released 2010. IBM SPSS Statistics for Windows, Version 19.0. Armonk, NY: IBM Corp.). Continuous variables were reported as means ± standard deviation (SD). Medians with interquartile ranges (IQRs) were used to describe non-normally distributed variables. Finally, categorical variables were presented as numbers and percentages. We used Pearson’s correlation coefficient to evaluate the associations between two continuous variables. Correlations between ordinal and continuous variables were measured based on Spearman’s rho correlation coefficient. A linear regression model was applied to control and adjust the effects of possible confounders and prevent the results from becoming biased.

Furthermore, we determined the power of anti-MCV titer in the prediction of reduced LVEF (LVEF<50%) using the receiver operating characteristic (ROC) curve analysis. This was done by plotting sensitivity against (1 − specificity) and calculating the area under the curve (C-statistic) as the prediction power. We also calculated a cut-off value for anti-MCV to discriminate between reduced (LVEF<50%) and preserved LVEF (LVEF≥50%). The value that was correlated with the highest sensitivity and specificity was used as the cut-off value.

## Results

We studied 135 patients with RA (mean age = 52.3 years; 111 (82.2%) females) (Table 1). Their baseline characteristics and echocardiographic parameters are presented in Table 1. Most of the data were complete; however, IVRT is missing for 12 (8.9%) cases (Table 1). All included patients received 2.5–7.5 mg of prednisolone per day.

**Table 1:** Baseline characteristics and echocardiographic parameters of participants

Characteristic	Value^a^	Missing data^a^
*Demographics*		
Age (year)	52.3 ± 11.9	0 (0%)
Female sex	111 (82.2%)	0 (0%)
Female to male ratio	4.6	0 (0%)
Height (meter)	1.58 ± 0.08	0 (0%)
Weight (kilogram)	71.0 ± 13.8	3 (2.2%)
Body mass index (kg/m^2^)	28.6 ± 5.3	3 (2.2%)
Duration of disease (year)	9.7 ± 9.4	0 (0%)
DAS-28/ESR	2.1 ± 0.4	1 (0.7%)
SDAI	2.7 ± 0.7	1 (0.7%)
ACR functional class	1 [1]	1 (0.7%)
Dyslipidemia	30 (22.2%)	0 (0%)
Diabetes mellitus	27 (20.0%)	0 (0%)
Hypertension	35 (25.9%)	0 (0%)
Framingham score (%)	2.7 ± 4.1	0 (0%)
*Medications*		
Prednisolone	135 (100%)	0 (0%)
Hydroxychloroquine	128 (94.8%)	0 (0%)
Methotrexate	125 (92.6%)	0 (0%)
Leflunomide	36 (26.7%)	0 (0%)
Sulfasalazine	30 (22.2%)	0 (0%)
*Laboratory data*		
RF (IU/mL)	0 [2]	0 (0%)
ACPA (IU/mL)	91.5 ± 115.9	0 (0%)
Anti-MCV (IU/mL)	150.1 ± 290.0	0 (0%)
Total cholesterol (mg/dL)	182.6 ± 33.9	0 (0%)
Low-density lipoprotein cholesterol (mg/dL)	106.9 ± 31.1	0 (0%)
High-density lipoprotein cholesterol (mg/dL)	50.3 ± 11.9	0 (0%)
Triglyceride (mg/dL)	137.6 ± 57.4	0 (0%)
*Echocardiographic indices*		
LVEF (%)	57.0 ± 5.4	0 (0%)
PAP (mmHg)	26.7 ± 7.8	10 (7.4%)
E velocity (m/second)	0.8 ± 0.2	0 (0%)
E/e’ ratio	9.8 ± 4.9	3 (2.2%)
TAPSE (mm)	23.2 ± 3.1	5 (3.7%)
IVRT (millisecond)	125.4 ± 19.1	12 (8.9%)
RVSm (cm/s)	12.3 ± 1.8	10 (7.4%)

*ACPA* Anti-citrullinated protein antibody, *ACR* American college of rheumatology, *Anti-MCV* Anti-modified citrullinated vimentin, *DAS-28/ESR* Disease activity score-28/erythrocyte sedimentation rate, *IVRT* Isovolumic relaxation time, *LVEF* Left ventricular ejection fraction, *PAP* Pulmonary arterial pressure, *RF* Rheumatoid factor, *RVSm* Right ventricular systolic motion, *SDAI* Simple disease activity index, *TAPSE* Tricuspid annular plane systolic excursion

^a^Data are reported as Mean ± SD, Median [IQR], or Number (Proportion%)

Among all evaluated echocardiographic variables, E velocity showed a statistically significant inverse correlation with RF (Spearman’s rho coefficient = − 0.223, *P* = 0.009). Furthermore, ACPA and anti-MCV demonstrated similar associations with LVEF (Pearson’s coefficient = − 0.201, *P* = 0.019; Pearson’s coefficient = − 0.322, *P*<0.001, respectively). We did not find any other statistically significant correlations between RF, ACPA, or anti-MCV with assessed echocardiographic indices (Table 2).

**Table 2:** Association of echocardiographic parameters with auto-antibodies

Parameter		RF	ACPA	Anti-MCV
LVEF (%)	Coefficient	−0.161 ^a^	−0.201 ^b^	−0.322 ^b^
	P	0.061	0.019	<0.001
PAP (mmHg)	Coefficient	0.060 ^a^	−0.036 ^b^	0.077 ^b^
	P	0.504	0.692	0.393
E velocity (m/second)	Coefficient	−0.223 ^a^	−0.011 ^b^	0.083 ^b^
	P	0.009	0.903	0.336
E/e’ ratio	Coefficient	0.128 ^a^	0.045 ^b^	0.097 ^b^
	P	0.144	0.610	0.268
TAPSE (mm)	Coefficient	−0.142 ^a^	−0.092 ^b^	0.078 ^b^
	P	0.106	0.296	0.376
	P	0.311	0.546	0.716
IVRT (millisecond)	Coefficient	0.028 ^a^	0.058 ^b^	0.128 ^b^
	P	0.759	0.523	0.157
RVSm	Coefficient	0.008 ^a^	−0.021 ^b^	0.012 ^b^
	P	0.927	0.817	0.897

*ACPA* Anti-citrullinated protein antibody, *Anti-MCV* Anti-modified citrullinated vimentin, *IVRT* Isovolumic relaxation time, *LVEF* Left ventricular ejection fraction, *PAP* Pulmonary arterial pressure, *RF* Rheumatoid factor, *RVSm* Right ventricular systolic motion, *TAPSE* Tricuspid annular plane systolic excursion

^a^Spearman’s rho correlation coefficient

^b^Pearson correlation coefficient

To consider possible confounding effects, a simple linear regression model was fitted to the data to predict LVEF based on anti-MCV, ACPA, and all possible confounders, including the Framingham score. A significant regression model was constructed (F (2, 128) = 12.429, *P*<0.001) with an R-square of 0.163. In this model, anti-MCV and age were significant predictors of LVEF; nonetheless, the association between LVEF and ACPA did not remain significant after the adjustment for possible confounders (Table 3).

**Table 3:** Linear regression analysis for prediction of LVEF

Variable	Multivariate analysis	
	Coefficient (95% confidence interval)	P
Anti-MCV	−0.006 (− 0.009_ − 0.003)	<0.001
Age (year)	−0.111 (− 0.186_ − 0.037)	0.004
Constant	63.604 (59.577_67.632)	<0.001

*Anti-MCV* Anti-modified citrullinated vimentin

A ROC curve was plotted to predict reduced LVEF (LVEF<50%) based on the anti-MCV titer. The area under the curve, which represents the strength of the prediction, was 0.843 (95% confidence interval: 0.631_1.000, *P* = 0.002). An anti-MCV titer of greater than or equal to 547.5 IU/mL identified reduced LVEF with a sensitivity of 85.7% and a specificity of 93.0%.

## Discussion

We have investigated the correlation between autoantibodies and echocardiographic findings in patients with RA who were asymptomatic for CVDs. To more accurately estimate the influence of autoantibodies per se on cardiac imaging findings, the confounding effects of demographic features, medications, atherosclerosis, and disease duration were eliminated by multivariate regression analysis. In addition, the cumulative undesirable effects of a prolonged inflammatory state of RA were also considered in the analyses using the ACR functional class of RA. Our findings showed that the anti-MCV antibody and RF titers were associated with reduced LVEF and E velocity, respectively; however, we did not identify any significant correlation between echocardiographic findings and ACPA level.

Anti-MCV is an antibody that protects against citrullinated vimentin. Therefore, it is categorized as an ACPA. It is primarily identified as a diagnostic marker in RA, but its sensitivity seems not to be significantly different from non-specific ACPA [13]. However, some added value of its concomitant measurement with ACPA was proposed to augment the sensitivity of early RA diagnosis [14]. Nevertheless, limited studies have gone beyond the diagnostic value of anti-MCV antibody to investigate its clinical value in RA.

Notably, it was demonstrated that anti-MCV antibody level was associated with inflammatory markers (ESR and C-reactive protein), disease activity, and carotid intima-media thickness (CIMT) in treatment-naïve RA cases [15]. In addition, the anti-MCV antibody titer was diminished after treatment commencement, and its changes were correlated with changes in cardiovascular risk factors including CIMT, total cholesterol, low-density lipoprotein cholesterol/high-density lipoprotein cholesterol, interleukin 6, and tumor necrosis factor-alpha [15]. Nonetheless, no association was identified between ACPA changes and the markers mentioned above. This finding could indicate the pathogenic role of anti-MCV antibodies in early precocious atherosclerosis among patients with RA [15].

The negative association between anti-MCV antibody titer and LVEF could be justified by a certain hypothesis. Giles and colleagues found a significantly higher level of citrullinated proteins in the interstitium of the myocardium of patients with RA than in healthy individuals [10]. Moreover, they demonstrated that myocardial fibrosis was associated with high citrullination scores in RA myocardium slides [10]. Surprisingly, the citrullination of sarcomeric proteins, including vimentins, was demonstrated in the myocardium of patients with heart failure [16], and this process can significantly diminish the activity and contraction of cardiac sarcomeres [6]. Taken together, these observations provide strong but still inconclusive evidence that antibodies against citrullinated cardiac proteins induce inflammatory and destructive damage to the myocardium and further modify the cardiac function in patients with RA. This hypothesis is prominently in line with the association between anti-MCV titer and LVEF.

The results obtained in the present study did not reveal any correlations between ACPA level and echocardiographic variables. Similar to our findings, several studies have also reported that there is no significant correlation between ACPA and LV systolic failure as defined by reduced LVEF and LV geometrical changes toward concentric remodeling [17–19]. In addition, most previous studies did not support the association of ACPA with diastolic dysfunction and LV mass index [20–23]. However, Logstrup et al. found a correlation between LV systolic dysfunction and ACPA in drug-naïve RA patients with normal LVEF after a two-year follow-up [24]. They assessed LV function by conventional echocardiography and speckle-tracking echocardiography and demonstrated that patients with persistently elevated ACPA over 2 years exhibited smaller improvements in S’ and worsened global longitudinal systolic strain (GLS) [24]. Changes in GLS and ACPA titer were also significantly correlated during the follow-up period [24]. In summary, it appears that this discrepancy comes from the different antibodies measured by each ACPA laboratory kit. We hypothesize that certain ACPAs that target citrullinated proteins of the myocardium have cardiotoxic effects, whereas the details of antibody subgroups have not been identified in previously mentioned studies.

The present study was subject to a number of potential methodological weaknesses. Due to the cross-sectional design of the study, we could not assess the causal relationship between the anti-MCV antibody titer and diminished cardiac systolic function. This issue warrants future prospective studies to address this shortcoming. Moreover, we had missing data (up to 8.9%) for some echocardiographic indices. In addition, we did not assess some echocardiographic parameters that are necessary to determine the exact grading of diastolic dysfunction, like left atrial size and index. Thus, we could not reliably examine the association between autoantibodies and cardiac diastolic dysfunction. Moreover, we did not evaluate serum markers of heart failure, such as N-terminal pro-B-type natriuretic peptide, which can be helpful. In addition, participants’ long-term use of medications might have affected our findings. This potential problem could be addressed by calculating the cumulative dose. The paucity of data regarding this issue is considered as another limitation of this study. Future prospective controlled studies that address the shortcomings of the present work are strongly encouraged.

## Conclusions

To the best of our knowledge, this is the first study that has attempted to determine the association between anti-MCV antibody level and the systolic function of the heart in patients with RA. Although the inverse correlation of anti-MCV antibody titer with LVEF may have pathophysiologic justifications, future prospective studies will determine the role of this antibody in the screening and early detection of cardiac systolic dysfunction in RA patients.

## Supplementary information

**Additional file 1.** Analyzed Data.

## Data Availability

The dataset supporting the conclusions of this article is included within the article and its additional file.

## Abbreviations

ACPA: Anti-citrullinated protein antibody
ACR: American college of rheumatology
Anti-MCV: Anti-modified citrullinated vimentin
CIMT: Carotid intima-media thickness
CVD: Cardiovascular disease
DAS-28/ESR: Disease activity score-28/erythrocyte sedimentation rate
EULAR: European league against rheumatism
GLS: Global longitudinal systolic strain
IQR: Interquartile range
IVRT: Isovolumic relaxation time
LVEF: Left ventricular ejection fraction
PAP: Pulmonary arterial pressure
RA: Rheumatoid arthritis
RF: Rheumatoid factor
ROC: Receiver operating characteristic
RVSm: Right ventricular systolic motion
SD: Standard deviation
SDAI: Simple disease activity index
TAPSE: Tricuspid annular plane systolic excursion
TTE: Transthoracic echocardiography
